# Qualitative research in suicidology: a systematic review of the literature of low-and middle-income countries

**DOI:** 10.1186/s12889-023-15767-9

**Published:** 2023-05-19

**Authors:** Humayun Kabir, Sarah Wayland, Myfanwy Maple

**Affiliations:** 1 Department of Sociology, University of Dhaka, 1000 Dhaka, Bangladesh; 2 School of Health, Faculty of Medicine and Health, University of New England, 2351 Armidale, NSW Australia

**Keywords:** Low-and middle-income countries, Suicide, Causes of suicide, Suicide prevention, Qualitative research, Systematic review

## Abstract

**Background:**

Suicide is more prevalent among disadvantaged, discriminated, and marginalised people with the majority of global suicide deaths occurring in the low-and middle-income countries (LMICs). This can be attributed to sociocultural contexts and exacerbated by access to limited resources and services that can assist with early identification, treatment, and support. Accurate information on the personal experiences of suicide is lacking, as many LMICs legislate that suicide is illegal.

**Methods:**

This study aims to review the qualitative literature to explore the experiences of suicide in LMICs from the first-person perspective. Following the PRISMA-2020 guidelines, the search for qualitative literature published between January 2010 and December 2021 was undertaken. A total number of 110 qualitative articles from 2569 primary studies met the inclusion criteria. Included records were appraised, extracted, and synthesised.

**Results:**

The results provide lived experience insight into suicide from those living in LMICs, including understanding variations of the causes of suicides, the impacts on others exposed to suicide, existing support systems, and prevention measures to reduce suicide among LMICs. The study offers a contemporary understanding of how people in LMIC experience suicide.

**Conclusions:**

The findings and recommendations are derived from the similarities and differences within the existing knowledge base that is dominated by evidence from high-income countries. Timely suggestions for future researchers, stakeholders, and policymakers are provided.

**Supplementary Information:**

The online version contains supplementary material available at 10.1186/s12889-023-15767-9.

## Introduction

The current annual global suicide death rate of 11.4 per 100,000 population [1] tells us that suicide is a significant public health concern. However, the lack of timeliness and accuracy of data reporting indicates that these rates are likely to be higher compared to the reported data [1]. Several broad-scale sociocultural factors impact under-reporting including criminalising suicide, cultural stigma of suicidal deaths, misclassification of suicidal deaths as accidental deaths, and lack of or poor suicide reporting systems in low- and middle-income countries (LMICs) [2, 3]. Without resolution of these challenges, it is not possible to accurately understand the true extent of suicide in LMICs. Nevertheless, suicide is considered a significant public health concern [4–12] and it has always been a major public health concern worldwide [13–18]. However, WHO data on mortality indicates that 75.5% of the world’s suicides occur in LMICs [1].

The causes and frequency of suicidal behaviour and suicide death clearly signify the importance of studying suicidology in LMICs with the recent interest in these areas increasing [12, 19–25]. Poverty [26], sociocultural and religious challenges [19, 20, 27], family factors [5, 12], physical disability [28]; shame and stigma associated with mental health illnesses, social challenges, and the impact of being disconnected from family members [23] are all found to play important roles in suicide across LMICs. Interpersonal factors such as suicide attempts being seen as attention seeking [22, 29] and the impact of relationship breakup [21], as well as traditional masculinity norms as a barrier to help-seeking [2] potentially, contribute to suicidal behaviours in LMICs. Recent studies have emphasised mental disorders (MDs) [30, 31] as well as mental and substance use disorders [32] as the mounting risk factors for suicide. A recent study focused on the lack of treatment coverage and underdiagnosis status of major depressive disorders in LMICs [33], highlights the ways in which support of mental health conditions create potential threats for deaths by suicide.

However, given the broad and interpersonal sociocultural norms influencing suicidal behaviours in LMIC, coupled with suicide being illegal in many jurisdictions, delving into the personal experiences of suicide is challenging. Nevertheless, in so doing, deep insights into suicide – and thus suicide prevention – can be gained.

Hjelmeland and Knizek [34] argue that quantitative methodologies are limited and do not allow a full examination of the complexity of suicide. Such research design results in a greater understanding of the underlying causes of suicidal behaviour while remaining reliant on linear cause-and-effect relationships [34]. In addition, Wilde [17] argued that as long as the measurements in quantitative methods (undiscovered statistical techniques, unnecessary variety, unproven validity, and insignificant results) to study suicidology are not addressed, qualitative research remains far more indicative of the lived experience of suicide in LMICs than quantitative research. Moreover, qualitative research, gives voice to people who experience suicidal behaviour [35] and provides critical insights into prevention and intervention that can influence or disrupt suicidal behaviours. Despite giving the voice to people with lived experience of suicidal behaviour [36], qualitative researchers propose different pathways to minimise the gap between real world action and insights from qualitative research [37, 38]. While initiating the search to find relevant research, we found no systematic review was conducted to understand the LMIC experience suicides along with the causes and consequences. It is within this context that a systematic review was conducted with the aim of understanding the contribution that qualitative research has made to the contemporary suicidology understanding of suicide in LMICs. To do this the causes, means, consequences, and prevention strategies of suicidal behaviour in LMICs were reviewed. Thus, the question driving this research is: “What is the role of qualitative research in understanding causes, consequences, and prevention strategies of suicidal behaviour in LMICs?”. This systematic review focused only on the studies that collected data on first person perspectives. This was done to ensure the best possible representation of suicide vulnerable participants [39], whose voice are largely silent in literature [24], and to make a true claim about the causes of suicide [36].

## Methods

In line with Widger [40], we emphasis on how suicidology, as a social practice seeks to perpetuate norms, values, and traditions. It articulates what suicide actually is, what the aims of suicide research should be, and how this ought to be achieved. Through this definition, professional suicidologists propose a particular way of ‘seeing and doing’ the study of suicide. In this review, we have used the term ‘suicidology’ to understand suicides along with causes and consequences, as well as prevention, in LMIC context.

### Protocol and registration

The systematic literature review was performed in line with the Preferred Reporting Items for Systematic Reviews and Meta-analyses (PRISMA)-2020 guidelines, which is a common practice to identify both qualitative studies [41–45] and quantitative studies [30, 31, 33]. The review protocol was registered with PROSPERO in 2021 (Registration Number: CRD42021250857).

### Inclusion and exclusion criteria

The search strategy sought to understand the peer-reviewed empirical research. The following criteria were used to determine the eligibility of studies: (1) primary research that focussed first-person voice of participants who have experienced suicidal thoughts, survived a suicide attempt, cared for someone through a suicidal crisis, or been bereaved by suicide; (2) location within LMICs as according to the World Bank (WB) at the commencement of the study period (2010) [46]; (3) qualitative research (conducted by using qualitative methodologies such as interviews, storytelling, yarning, or focus groups and provides evidence of the experiences and perspectives of those with a lived experience of suicide), see Table 1 below for details; and (4) the research which was conducted between January 2010 and December 2021, and was peer-reviewed and published in English.

**Table 1:** Methods used and corresponding definitions

Methods used	Definition
Semi-structured interviews [17, 21, 23]	A good opportunity for the participants to talk about their issues (such as suicide attempts) without any restriction caused by the closed question of questionnaires
Semi-structured in-depth interviews [12, 14–16, 22, 33, 37, 38]	-Detailed and rich understanding participants’ perspectives/views, listening and observing them closely, and noting their body language and tone of voice
Narrative interviews [13, 36]	Uses of open-ended narratives to understand how participants engage in suicidal behaviour themselves explain their actions in actual concrete events in space and time

The following exclusion criteria were applied: (1) quantitative and mixed-methods research; (2) studies that focused on organisational approaches to support for individuals or communities and did not articulate the first-person voice of those experiencing a lived experience of suicide; (3) studies that were in languages other than English or with a location outside of LMICs (Non-English studies were not included in the initial searches); (4) research employing a hypothesis-deductive or experimental methodology focused on the explanation of suicidology, for example, randomised controlled trial studies, (neuro) biological studies, epidemiological studies and discursive papers, opinions, or editorials; and (5) books, dissertations, and unpublished data or manuscripts were not included. The review excluded mixed-methods studies given the study sought to focus only on qualitative studies. This then excluded qualitative components of the mixed-methods research.

### Literature search

A three-step search strategy was implemented to ensure all relevant literature related to the topic under investigation was identified. The three steps include: (1) J.D. undertook a limited search using EBSCO-Host and ProQuest utilising keywords (suicide, suicidology, lived experience, qualitative research); (2) the keywords from over 200 returns were then reviewed with specific keywords highlighted for possible inclusion in a revised strategy; and (3) the search was then re-designed in consultation with the University of New England (Australia) health librarian to develop a full search strategy. The full literature search was completed by using six electronic databases including ProQuest, EBSCO Host, Pub-Med, Web of Science, Google Scholar, and Sage Journals. The searches were conducted and recorded by J.D. and M.M. on two separate university library systems to ensure the search was replicable. In addition, H.K. conducted a rigorous hand-searching of titles of the final included studies. Search terms included the following keywords: su((suicide OR suicidology OR suicides OR suicidal OR suiciding) AND (qualitat* OR “qualitative research design” OR narrative OR phenomenology OR interviews OR “mixed method” OR “mixed methods”) AND (“lived experience” OR prevention OR intervention OR postvention)). The limiters applied were as follows: “Published between January 2010 and December 2021”, “Peer Reviewed”, and “Scholarly Journals”. We adopted the search to identify the research published in twelve years period of time. It is a very common practice to add an average of ten years period in the recent published systematic reviews [see 31, 43, 44]. Researchers usually extend the time period if there is lack of available studies in 10 years period of time. The detailed search strategy is displayed in Appendix S1.

### Study selection

A total of 2569 studies were identified from database searches. All search results were collated and uploaded into the citation management tool Endnote X9 (2020) and 1161 duplicates were identified automatically and removed. M.M. and J.D. met to pilot their screening process against the agreed inclusion/exclusion criteria. Records were then uploaded into Covidence systematic review system [47] to complete the title and abstract and full-text screening. Covidence automatically identified and removed an additional 50 duplicates. Then, 1358 studies were screened independently by M.M. and J.D. who met on four occasions to discuss any recorded disputes which were then resolved through discussion. On completion of the full-text screening, there were 110 studies that met inclusion criteria. A review meeting with M.M., J.D., H.K. and S.W. took place and the decision was made to limit studies to LMICs perspectives and to exclude mixed-methods studies and the studies that did not prioritise the first-person voice of people with lives experience of suicide. Prior to the location of the study being identified within each study based on WB classification for LMICs in 2010 [46]. M.M. and H.K. identified the location of each study, resulting in a further 90 studies being excluded. During this process, a further nine studies were excluded due to the lack of primary data or not presenting first-person voices. Two articles were included from the rigorous hand-searching of titles of the final included studies.

**Fig. 1:**
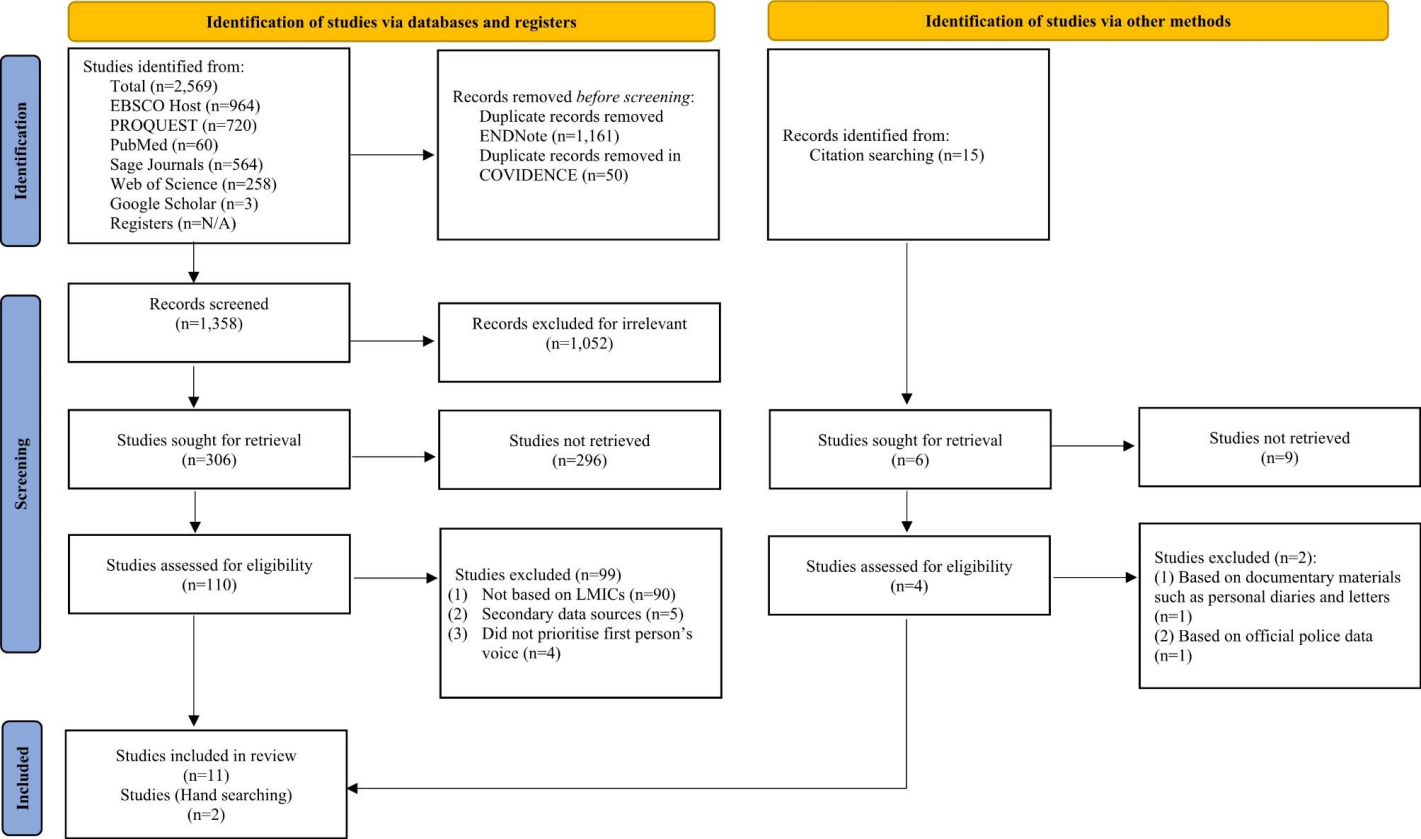
PRISMA flowchart

The studies that met the inclusion criteria (n = 13) were then uploaded into the JBI SUMMARI systematic review programme [48] for Quality Appraisal, Data Extraction and Synthesis. It is worth noting here that we have explored a good number of previously published reviews that found small number of articles after the rigorously following PRISMA guidelines, such as Usher et al. [41] (n = 8); Kaniuka and Bowling [42] (n = 11); Durkin et al. [45] (n = 11); Fatema et al. [43] (n = 16). In addition, we had to exclude 99.95% of the research due to applying the inclusion/exclusion criteria. The full selection process is outlined in the PRISMA flow chart (Fig. 1) [49].

### Quality assessment

Authors H.K. and M.M. critically appraised the studies. The Critical Appraisal Skills Programme [50] tool was used to assess the quality of the included studies. This tool is appropriate for qualitative reviews and has been used in previously published systematic reviews of similar literature [43, 44]. The CASP checklist contains ten items with a maximum of one score for each item. The quality was defined as ‘strong’ if the article scored 9–10, ‘moderate’ if it scored 5–8, and ‘weak’ if the article scored 1–4 [44]. The quality assessment procedure represents eleven of thirteen studies as ‘strong’ and the remaining two as ‘moderate’ (Table 2). The reported limitations of the two studies assessed as moderate were: Osafo et al. [51]–‘consideration of ethical issues’ and ‘clear statement of findings’; and Amin et al. [22]–‘appropriate research design’ and ‘relationship between researcher and participants have been adequately considered’.

**Table 2:** Assessing the quality of the papers

Assessing the quality of the studies through using CASP (Critical Appraisal Skills Programme) tool (yes = 1, no = 0)												
Author(s) & Year of publication	1. Clear statement of research aims	2. Appropriate methodology	3. Appropriate research design	4. Appropriate recruitment strategy	5. Justification of the way of data collection	6. Relationship between researcher & participants has been adequately considered	7. Consideration of ethical issues	8. Rigorous data analysis	9. Clear statement of findings	10. Value of research	Scores attained (Out of 10)	Ratings (1–4 = weak, 5–8 = moderate, 9–10 = strong)
Mathew et al. [12]	1	1	1	1	1	1	1	1	1	1	10	Strong
Akotia et al. [36]	1	1	1	1	1	0	1	1	1	1	9	Strong
Acheampong & Aziato [22]	1	1	1	1	1	1	1	1	1	1	10	Strong
Akotia et al. [13]	1	1	1	1	1	1	1	1	1	1	10	Strong
Asare-Doku et al. [17]	1	1	1	1	1	1	1	1	1	1	10	Strong
Osafo et al. [33]	1	1	1	1	1	1	0	1	0	1	8	Moderate
Shamsaei et al. [14]	1	1	1	1	1	1	1	1	1	1	10	Strong
Azizpour et al. [23]	1	1	1	1	1	1	1	1	1	1	10	Strong
Rezaie et al. [21]	1	1	1	1	1	1	1	1	1	1	10	Strong
Sukhawaha et al. [15]	1	1	1	1	1	1	1	1	1	1	10	Strong
Wang et al. [37]	1	1	1	1	1	1	1	1	1	1	10	Strong
Amin et al. [16]	1	1	0	1	1	0	1	1	1	1	8	Moderate
Medina et al. [38]	1	1	1	0	1	1	1	1	1	1	9	Strong

### Data extraction

A standard data extraction form was used [42, 52], which included: the article’s general information (title, author, publication year, phenomenon of interest/objectives), methodology (method, setting, geography, cultural, participants, data analysis) findings/outcome information, and authors conclusions. These criteria were included in the JBI qualitative data extraction form. H.K. independently extracted all data and then reviewed by M.M. to retain the accuracy and consistency of data. This extraction summary is reported in Table 3.

**Table 3:** Reviewed articles summary

Authors Country	Methodology	Phenomena of interest	Factors associated with suicide	Findings/Outcomes	Quality
Mathew et al. [12] India	-Qualitative -Purposive sampling -In-depth interviews -Each interview lasted about 30 min -Participants were recruited from in-patient wards of a tertiary care medical hospital -Sample size: 22 (Female: 11; Male: 11) -Age: 13–29 years -Thematic analysis was performed	Explored the family factors contributing to the suicide attempt	-Lack of family support -Low socioeconomic factors -Hostile environment within the household environment	-Lack of family support and a volatile home environment (i.e., conflictual interactions between family members, parental conflicts and separation, conflict with a sibling or other members of the family, and marital disharmony) leads to suicidal attempts among adolescents and young adults -Socioeconomic factors (such as financial issues, superstitious beliefs, disturbing neighbourhoods, interpersonal issues, and the stigma of having a mental illness) may lead to suicidal behaviour	Strong
Akotia et al. [36] Ghana	-Qualitative - Purposive sampling -Narrative interviews -Interviews ranged from 12–80 min - Participants were recruited from five hospitals and clinics in Accra, the capital of Ghana - Sample size: 30 (Female: 18; Male: 12) -Age: 18–46 years -Interpretative Phenomenological Analysis was performed	Examined the role played by religion in the experiences of persons who attempted suicide	-Religion is related to suicidal behaviour	-Participants acknowledged God as the owner of life and death -Suicide attempts are considered as own responsibility and a crime against God -Participants experienced guilt and tried to restore the relationship with God by asking for forgiveness -Few of the participants seemed to understand God as a tyrant as he did not fulfil his promises even though they had fulfilled their religious duties	Strong
Acheampong & Aziato [22] Ghana	-Qualitative -Purposive sampling -In-depth interviews -Interviews lasted 30–40 min -Participants were recruited from a major rehabilitation centre located in Accra, the capital city of Ghana -Sample size: 12 (Female: 12) -Age: 21–57 years -Data were analysed inductively using the content analysis technique	Explored suicidal ideations and coping strategies of mothers living with physical disabilities	-Social isolation -Separation from family and friends -Feeling of being discriminated	-Discrimination makes people living with disability feel isolated. -Eventually, isolation may lead to a sense of separation from the masses and separation may mean attempting suicide and leaving this world -Coping strategies to avoid suicide attempts include the reason for having children, self-motivation, counselling, assistance from relatives, and prayer	Strong
Akotia et al. [13] Ghana	-Qualitative -Purposive sampling -Narrative interviews -The interviews lasted from 30 to 40 min (with one exception, 15 min) -Participants were recruited from five hospitals in Accra, the capital of Ghana -Sample size: 30 (Female: 18; Male: 12) -Age: 18–46 years -Interpretative Phenomenological Analysis was performed	Examined the reasons for suicide attempts among patients in Ghana	-Existential struggles -Lack of social support -Supernatural beliefs -Abandonment	-Lack of support, shame, abandonment, existential struggles, and supernatural reasons were reasons identified as contributing to the suicide attempt -Gender differences in the reasons for attempting suicide: (1) men resorted to suicidal behaviour when faced with shame, existential struggles, and supernatural reasons; (2) Women tried to kill themselves when they felt they lacked social support or faced abandonment.	Strong
Asare-Doku et al. [17] Ghana	-Qualitative -Purposive sampling -Semi-structured interviews -Each interview lasted 40–60 min -Participants were recruited from the Psychiatry Department of Korle-Bu Teaching Hospital located in Accra, within the Greater Accra Region of Ghana -Sample size: 10 (Female: 4; Male: 6) -Age: 25–62 years -Interpretative Phenomenological Analysis was performed	To understand the experiences of the families of attempt survivors and how they cope with the aftermath of the attempt	-Shame to being survived the suicide attempt -Lack of institutionalized professional support for the suicide survivors	- Negative experiences and reactions towards the suicide attempts: experiencing shame and stigma, reactive affect, and surviving the stress of the attempt - Participants were experiencing shame with feared stigma following the suicide attempt of their relatives - Attempt survivor families did not receive much help following the attempt. This might reflect both self-stigma (which in some of the cases discouraged informants from seeking help), lack of credible institutionalized professional support and the generalized negative attitudes toward suicide in Ghana	Strong
Osafo et al. [33] Ghana	-Qualitative -In-depth interviews -Participants were recruited from a small village in the Eastern Region of Ghana with a population of about 500 inhabitants -Sample size: 10 (Male: 10) -Age: 30–41 years -Thematic analysis was performed	To understand the experiences of suicidal persons in Ghana	-Social taunting, hopelessness, and partner’s infidelity. -Suicidal persons reported stigma expressed through physical molestation and social ostracism, which left them traumatized	-Suicidal persons reported stigma expressed through physical molestation and social ostracism, which left them traumatized -Participants’ sense of hopelessness was linked to despair arising from their present living conditions, such as lack of job and hassles of living with a chronic illness -The analysis has further shown that the life of a suicidal person in the rural context is a traumatic experience. Community stigma increased their pain, causing further trauma -Coping strategy includes social support from relations, religious faith, and use of avoidance.	Moderate
Shamsaei et al. [14] Iran	-Qualitative -Purposive sampling -In-depth interviews -Interviews ranged from 40 to 60 min -Participants were recruited from the Farshchian Psychiatric Hospital in Hamadan, Iran -Sample size: 16 (Female: 4; Male: 12) -Age: 19–57 years -Interpretative Phenomenological Analysis was performed	-Explored the lived experience of attempted suicide with the phenomenology approach	-Social isolation/lack of sense of belongingness -Social and economic factors -Mental pain	-Participants have talked about suffering and problems that has different dimensions including mental pain, family, social and economic factors, and the need for understanding and loving -Suicide risk is most commonly associated with mental illness. Mental disorders play an overwhelming role in the increased risk of suicide -The levels of mental pain are associated with an increased risk of suicide -The sociocultural factors that affect suicide rates operate at many different levels including demographic characteristics, life stressors, coping skills, and economic status linked to suicide -Love and a sense of belongingness would decrease suicidal behaviour	Strong
Azizpour et al. [23] Iran	-Qualitative -Purposive sampling -Semi-structured interviews -Interviews lasted between 45 to 95 min - Participants were recruited from the Two teaching hospitals (referral centers for suicide attempters) in Ilam, Iran -Sample size: 7 (Female: 7) -Age: 20–37 years -Interpretative Phenomenological Analysis was performed	To understand the experience of women after suicide attempts	-Gender discrimination -Social pressure particularly on women	-Loved ones keeping an eye on them, i.e., families of suicide attempters had taken a very cautious approach toward them. Female suicide attempters were not allowed to be alone, and their families kept them under careful observation. For instance, “keeping suicide attempters under intensive supervision” made them feel as though their privacy rights were threatened by this careful observation. -Rain of love, i.e., families were worried about the possibility of the women’s sudden death resulting from suicide. This fear led to the emergence of the following reactions: “emotional reactions,” “emotional support,” “financial support,” and “accepting any demand.” The “rain of love,” and the unreasonable acceptance of any request and demand by the women in particular, gave them a sense of satisfaction regarding their suicidal behaviours.	Strong
Rezaie et al. [21] Iran	-Qualitative -Purposive sampling -Semi-structured interviews -Interviews lasted between 20 to 60 min -Participants were recruited from the Kermanshah Imam Khomeini hospital, Iran -Sample size: 15 -Age: 22–50 years - Data were analyzed using the constant comparative method which is the Grounded Theory data analysis method	Explored motives for suicide by self-immolation in Kermanshah, Iran	-Depression -Chronic severe stress -Conflicts between family members	-Five categories of mental health problems, family problems, cultural context, self-immolation as a threat, and distinct characteristics of the method were explored as motives for attempting suicide by self-immolation. - Mental health problems including primary psychiatric disorders (depression) and adjustment psychiatric disorders (such as an impulsive act to self-immolation were reported as a reaction to chronic or severe stress seems more important) were among the motives for attempting suicide by self-immolation - Results also show that existed conflicts between the family members of self-immolated patients, especially wives–husbands, and parents–children problems were a mentioned motive for attempting self-immolation	Strong
Sukhawaha et al. [15] Thailand	-Qualitative -Purposive sampling -In-depth interviews -Interviews ranged from 45 to 60 min -Sample size: 18 -Content analysis was performed	To describe attempted suicide triggers in Thai adolescents	-Unwanted Pregnancy -Harsh familial relationships -Depression -Poverty and low socioeconomic status -Domestic violence -Alcoholic family	-Regarding attempted suicide, there is a gender difference: females are more engaged in the suicidal attempt but are less likely to complete suicide than males. -The problems of relationships and harsh criticisms expressed with anger by important people in their lives, whether they are family members (especially mothers) or lovers greatly influenced adolescent’s emotions and the decision to attempt suicide - Females in this study are disappointed in love and unwanted pregnancy, both of which lead to the decision to attempt suicide - Pregnancy is a risk factor among women and most women who attempted suicide during pregnancy in the first period were young with lower socioeconomic status who had not finished their schooling - In terms of mental health, problems including depression lead to attempted suicide which is also found in this study -Family context may be the predisposing risk factor to adolescents’ attempted suicide; poverty or low socioeconomic, living apart from parents, living with a single mother or stepfather, having alcoholic parents, domestic violence and dysfunctional family	Strong
Wang et al. [37] China	Qualitative -Purposive sampling -Semi-structured and individual in-depth interviews -Interviews lasted between 20 to 60 min -Participants were recruited from a tertiary referral hospital in China -Sample size: 15 (Female: 14; Male: 1) -Age: 22–50 years -Thematic analysis was performed	Explored the impact of inpatient suicides on nurses working in front-line, the patterns of regulation and their needs for support	-Being inpatient -Psychological health vulnerabilities	-Inpatients were highly likely to die by suicide; considered difficult to prevent -Nurses lacked the necessary suicide prevention skills -Psychological responses mainly included shock and panic, self-accusation or guilt, sense of fear, and frustration -The impacts on practice were stress, excessive vigilance, and burnout -Avoidance and sharing of feelings played key roles in the regulation patterns of nurses - High levels of emotional distress and negative impact on practice in nursing staff working in the general hospitals. Their patterns of regulation to cope with inpatient suicide should be improved	Strong
Amin et al. [16] Iraq	-Qualitative -Purposive sampling -Semi-structured in-depth interviews -Participants were recruited from urban and rural areas of Soran District, Iraq Sample size: 24 (Female: 24) -Age: 21–45 years - Data were analysed using conventional content analysis	Explored probable issues which might lead to self-immolation in young Kurdish Iraqi women		Causes of self-immolation are: -Not having control over personal life, and marital conflicts (Some participants revealed that they chose to do self-immolation to get rid of marital conflicts) -Seeking attention (they tried to seek others’ attention and send them a persuasive message. By using the bravado of fire, they want to voice their complaints, so that those who have shut their eyes and ears and ignored them for so many years can hear what they have to say) -Instilling guilt in the family members (Ignoring the personal and social rights of women in some societies, especially in rural ones, due to their dominant culture, is likely to lead to such consequences as mental problems and depression), and -Resentment toward male dominant community (Most Kurdish women’s rights activists believe that, in these regions, self-immolation and suicide are a kind of protest against the male-dominated society and the discriminations and limitations imposed by the father, brothers, and the husband’s family.)	Moderate
Medina et al. [38] Nicaragua	-Qualitative -Purposive sampling -In-depth interviews - Participants were recruited from León municipality, Nicaragua -Sample size: 12 (Male: 12) -15-24 years -Data were analysed with a grounded theory approach	To understand the pathways leading to attempted suicide of young men in León, Nicaragua	-Frustration -Traumatic life experience -Experience antagonistic relationships within the family atmosphere	-Overall, suicide attempts can be viewed as a response to frustration caused by negative structural conditions both in terms of material circumstances and unfulfilled normative expectations -In all cases, the decision to attempt suicide was found to be an expression of frustration with the present conditions of life. Combined with this was the traumatic influence of a troubled childhood within an unloving, unstable family -Traumatic life experiences, exposure to suicidal behaviour among close relatives and interpersonal conflicts all served as triggering factors further fuelled by alcohol and drug intake -Attention has been paid to the ambivalent and antagonistic relationships that the informants experienced within their own families from childhood onwards, and the subsequent inability to establish any meaningful relationships in later life	Strong

## Results

### Study characteristics

Table 3 presents a summary of the key characteristics of the 13 studies included in this review. The studies were conducted in Ghana (n = 5), Iran (n = 3), Iraq (1), China (n = 1), Thailand (n = 1), India (n = 1), and Nicaragua (n = 1). This systematic review includes Africa, the Middle East, North America, Southeast Asia, East Asia, and South Asian regions of the world. The total number of participants included in these studies was 221. Of these participants, 184 (female: 110; male: 74) had attempted suicide or experienced suicide ideation. In addition, 20 medical professionals and 17 close family members of people who had attempted suicide were also included in these studies.

The qualitative methodologies used in these studies included semi-structured/in-depth individual (n = 11) and narrative interviews (n = 2) to address their research aims. Data were analysed by utilising interpretive phenomenological analysis (n = 5), thematic analysis (n = 3), content analysis (n = 3), and grounded theory approach (n = 2). From these included studies, six common themes were identified: (1) social taboo or stigma around suicidal behaviour in LMICs; (2) factors affecting individuals’ suicidal behaviour of LMICs; (3) means of suicide: observed variations among LMICs; (4) gendered dimension of suicidal behaviour in LMICs; (5) impacts of suicidal behaviour/attempts; (6) solution focused strategies to suicidal behaviours in LMIC. These are explored below.

### Social taboo or stigma around suicidal behaviour in LMICs

The included literature identifies that suicidal behaviour is taboo and socially stigmatised in LMICs. This is evidenced by evidence from India [12], Ghana [19, 23, 51, 53], Iran [29], and China [54]. In addition to discussions regarding suicide, the act of suicide attempt is considered a crime, where for example, people who attempt suicide in Ghana are subject to criminal penalties [53].

Social stigma toward suicide was also noted. These responses were presented surrounding discussions about suicidality, where others referred to people as sinners, transgressors, or people who are antisocial [23]. Suicide survivors, as well as their family members, refer to being physically assaulted, verbally abused, and socially as well as culturally excluded [19, 23, 51, 53] in response to suicide attempting. For some, their society excluded their loved one from a proper burial if the person had died by suicide [53].

### Factors affecting individuals’ suicidal behaviour of LMICs

The root causes of suicidal behaviour among people in LMICs identified in the literature were; the impact of hostility in the family environment (parental conflicts and separation, marital disharmony, conflicts with siblings and other family members) [12], using attempting as a way to seek attention from family members [22, 29] living with financial crises/poverty and chronic illnesses [20, 51], superstitious/supernatural beliefs that create suicidal behaviours [19, 53], and the guilt of surviving from the previous suicide attempt [23].

In addition to these causes, those with mental health issues also had the burden of the stigma of being vulnerable to mental illnesses [12, 21, 22, 27] coupled with a lack of institutional support for mental illness [23, 53] Both of which were noted as risk factors for suicide attempts. The studies emphasised that mental illness, is mainly linked to depression or frustrated efforts [22, 27], shock and panic [54], experiencing ‘mental pain’ [20], and past traumatic experience [51, 55] are predominantly ignored in the LMICs further increasing the risk for people who suicide attempt. Lack of support from relatives and community for people identified as vulnerable (for example those identified as having a physical disability and people experiencing complex mental health conditions) was found one of the leading causes of suicide in the LMICs [28].

### Means of suicide: observed variations among LMICs

The studies included in the review all identified common means of suicide such as - poisoning, drug overdose/ingestion of the poison, hanging, stabbing, jumping from unsafe heights, traffic accidents, and self-immolation [16, 19, 20, 22, 28, 55]. Variations in the means of suicide have been observed among LMICs that are specific to these regions.

Poisoning (such as intentionally consuming poisons such as detergent, acid, etc.) as well as drug overdoses were found to be the most common method of suicide attempts among the Ghanaian people [19, 28, 51]. People in Iran and Iraq present with suicide attempts that include self-harming activities (such as cutting and stabbing) – in addition to incidents such as hanging, jumping from significant heights, and self-immolation [20, 22] also featured. Literature relating to Nicaraguan people referred to the excessive use of alcohol and drugs contributing to suicide death [55].

### Gendered dimension of suicidal behaviour in LMICs

There are important gendered differences regarding causes of suicide, thoughts, behaviours, and attempts in LMICs.

#### Experiences of suicide involving women

The literature identified that women were at risk of suicide death when they lacked social support and experienced relationship abandonment [19]. In addition, the literature also explores the excessive social pressure and restrictions imposed on women in LMIC, to adopt traditional roles in the home [29]. In LMICs, women are mostly economically dependent on men, and when separation or divorce occurs, these impacts both financial survival and sense of self – with both increasing suicide risk [19].

In addition to social and relational factors, the literature also identifies domestic violence [21, 22] lack of intimate relationships as well as avoidance of unwanted pregnancies [21] as a suicide risk. Women in the Middle Eastern countries identified that restrictions in family and social life, requiring male family members’ company for outings [56, 57] create significant stress. Feelings of social isolation and discrimination can enhance risk factors for female suicide [29]. Further given the control of women in some locations, suicide was also reported as a giving voice in a culture that is silencing, for example, Amin et al. [22] identified that women’s suicide in the Middle East countries could be viewed as a protest to a male-dominated society and the discrimination and limitations imposed by the father, brothers, and the husbands’ family.

The literature also explores the impact of spirituality. Women in African countries were vulnerable to suicidal behaviour when they felt abandoned by God or were disappointed in their faith/God [19]. The literature also notes that women’s suicide attempts in LMIC are more prevalent, with women less likely to complete suicide than males [21]. Methods of suicide for women in LMIC refer to poisoning [28] and jumping from significant heights [20], indicating readily available, hard-to-control methods.

#### Experiences of suicide involving men

By comparison, the literature that referred to men’s experiences of suicide in LMIC differed. Community impacts such as living in poverty or identifying as being from a low socioeconomic status [12] were explored, as was the ongoing stress of unemployment [51]. Concerns relating to existential struggles with masculinity as well as stress connected to concerns about supernatural occurrences [19] were reported from male participants. Reference to men’s hegemonic masculine and masculine identity (explored as a sense of superiority, or perceived stoic strength) may discourage men in LMIC from seeking support when experiencing depression or experiencing suicidal ideation [55]. Methods of suicide were more likely to be the use of sharp instruments [20, 51], gunshots, and excessive use of alcohol and/or drugs [55].

### Impacts of suicidal behaviour or attempt

Suicidal behaviour impacts both the individual and their close family members, and questions deeply held beliefs. From a religiosity perspective, the central relationship with God as creating, or protecting, from suicidal behaviours was questioned. The studies identify that many disconnects from God as they consider God responsible for ongoing struggles. Akotia et al. [53] refer to suicide attempts in response to guilt from previous attempts and seeking a way to re-establish their relationship with God as a protective factor. Whereas others who have previously attempted, to seek distance from God despite continuing to perform their religious duties, some feel that their God did not fulfil his promises leading them to further suicidal activities [19]. This religious continuum centred on the need to be religiously obedient versus those who perceive their God creates the frustrations or failures that lead to suicide attempts that are worthy of further exploration [53].

Societally there was a lack of societal acceptance or compassion related to suicide attempts. Some studies showed that suicide survivors, along with their family members, can be culturally and socially excluded [19, 51]. For some suicide survivors their community responds with aggression; experiences of physical assault, verbal abuse, and social ostracism are perpetrated against the person who suicides attempted, or their family [51]. This may be a factor that further creates vulnerability for those who repetitively suicide attempt. Alternatively, other suicide survivors become the responsibility of family members [29] where acceptance of suicide attempters is offered unconditionally by families, as a protective strategy for subsequent suicide attempts.

### Solution-focused strategies for suicidal behaviours in LMIC

Strategies to reduce suicidal behaviours in LMIC were also presented in the retrieved records. Such strategies included early identification of mental health illnesses [12], easy access to mental health professionals [53], enhancing support systems for people with physical disabilities [28], promoting positive mental health and creating awareness to reduce mental health-related stigma [19]. The studies also referred to the integral role of family members, partners, and friends post-attempt [21, 55].

The screened studies identified a need for enhanced caregiver skills to create empathic interactions with those who suicide attempts [29]. Changes or modifications in cultural and religious contexts to reduce suicides in LMICs are required [19, 22, 23, 27]. These changes were characterised by potential adverse cultural constructions about life (such as all crises will be mitigated via death) [19], failure to resolve life challenges leading to a strong desire to die [27] meaning that inadvertently death is promoted as a solution, rather than resilience to the crisis or problem the person is experiencing.

Culturally sensitive programs (which permeate the attitudes, reactions, and coping strategies of LMIC lived experiences in that locale) to enhance suicide prevention strategies [23] were recommended and related to programs both for in-patient care, as well as in the community. Wang et al. [54] identified the need for medical professionals to acquire appropriate knowledge and skills in suicide prevention to reduce inpatient suicide risks. In addition, data from Ghana reinforced the need for religious institutions to be properly equipped with advanced level knowledge and training on mental health illnesses so that these institutions are involved in suicide prevention activities [23, 53].

## Discussion

Only two previous systematic reviews briefly examined the relationship between poverty and suicide [26] and the pattern of suicidal behaviour across the African continent [3]. Thus, this study is the first review (noting limitations mentioned in the later part in this review) the authors are aware of that seeks to investigate multiple factors affecting suicidology from the lived experience of those experiencing suicidal thinking or behaviours, and those who care for them in LMICs. Suicide prevention programs and strategies need to consider enhanced awareness of culture [58], gender inequalities, patriarchal system, and lack of financial autonomy for women in many LMICs.

The articles included in this systematic review indicate that knowledge about suicide prevention in LMICs is limited, yet the sociocultural influences on suicide are paramount to consider. These influences are explored through the causes, dimensions, means, and prevention strategies of suicidology in LMICs. The main suicide risk in LMICs relates to familial connections, poor socioeconomic conditions, and taboo/social stigma around mental health illnesses, cultural/superstitious beliefs, and lack of access or barriers to accessing mental healthcare. Suicide in LMIC requires a gendered lens, when identifying prevention activities, given the discrepancies between risk factors for women as opposed to men in LMIC. Women’s experience of violence, discrimination, and lack of freedom in the family and in the broader society results in suicide vulnerability among females. Patriarchal dominance within LMICs [3] contributes to women’s experiences as reactions against the sociocultural contexts that exist in LMICs. Conversely, men are pressured to adopt a ‘brave/real man’ image is paramount [2], which may also extend to the choice of means (firearm, cutting, alcohol). Previous studies found that men adopt more aggressive and deadly methods compared to women, thought to prove their strong masculine identity [2, 11]. Help-seeking is also challenged by societal norms, for example by men’s reported denial to seek mental healthcare or familial emotional support due to hegemonic masculine identity [2]. Here, a belief is held that confessing their struggles with distress or mental illness can destroy their social identity.

While for women, the pressure of social control was experienced also as limiting, yet more through interpersonal relationships with men exerting this control. Suicide was viewed as a way to be released from this control. The role of religion was also important across these studies. The outcome of these sociocultural factors plays out in suicide death rates recorded for males and females. Mars et al. [3] found that three to four more men die by suicide compared to women worldwide. While this ratio is reported for African countries (male: female ratio of 3:1) [3], across Asia more women die by suicide than men [59, 60], pointing to these important sociocultural contributors. Thus, suicide prevention programs and strategies need to consider enhanced awareness of culture [58], gender inequalities, patriarchal system, and lack of financial autonomy for women in many LMICs.

Low socioeconomic factors, cultural stigma, and superstitious beliefs were found dominant factors affecting suicidal behaviour in LMICs, not seen in the broader Western literature on suicide prevention. Risk factors commonly reported for suicide attempts in Western countries include mood disorders, personality disorders, negative emotions, addiction to alcohol/drugs, sexual orientation, isolation, etc. [61–66]. The results of the analysis allowed for the authors to engage in some broader comparison studies relating to suicide in Western countries and the role of qualitative research. What was highlighted was that in LMICs’ suicide is primarily related to basic needs and/or poor sociocultural and economic factors [67], while suicide in Western countries is mostly related to emotions, motivations, and capabilities to die by suicide [68, 69].

However, Neeleman et al. [70] conducted a study among 19 Western countries where they found that there were ecological associations between religion variables (such as religious belief and religious attendance) and suicide rates were prevalent among men and women. They showed that religious beliefs are associated with tolerance level of suicide which differed between men and women. For example, higher female suicide rates were associated with lower aggregate levels of religious belief and, less strongly, religious attendance [70].

High-income countries tend to adopt suicide prevention strategies that focus on broad public health measures coupled with individual service providers. Whereas there is a dearth relating to the approach to suicide prevention activities in LMICs to accurately identify its intent and purpose.

This study articulated first-person’s voices about suicidal ideation along with the causes of suicides in LMICs, from which new empirical research on this topic may be conducted in the future. The study identifies new knowledge relating to women in LMIC’s and their experiences of suicide, prompting the need to review data using a gendered lens. In addition, we highlighted variations in suicidology from region to region (Africa vs. Asia) although these regions vastly belong to LMICs. This study provided a snapshot of suicidology in the LMICs context which researchers and academicians may use as a reference point to continue further investigation and establish targeted research agendas.

### Limitations

There are inherent limitations as with all academic research and reviews. First, this study did not include research articles published before January 2010. Second, we did not consider reports or other grey literature sources, working papers, books and book chapters which may be published on the basis of primary data. Thus, there is a high chance that these sources may have retrieved additional results. Third, this study is limited by the design. Only papers with qualitative methods were included with the aim of understanding first-person accounts of suicide in LMICs. This limitation resulted in some important topics related to suicide being invisible, for example, seasonal variations were not mentioned, yet remained important in understanding suicidal behaviour in Western countries [71–74]. Fourth, other limitations were not due to the design of this review, but rather a limitation of the evidence. For example, most of the included studies were conducted in urban areas or hospital settings, thus no comment can be made relating to those who live outside of cities who may experience these – and other – factors that contribute to suicide. Fifth, restricting language to English for this review that focused on LMICs could potentially cause some studies to be overlooked. Sixth, the review excluded mixed-methods studies given the study sought to focus only on qualitative studies. Thus, we excluded qualitative components of the mixed-methods research. Lastly, since we considered the location within LMICs as according to the WB at the commencement of the study period (2010), we did not reflect if WB income level status for any LMICs changed between 2010 and 2021.

## Conclusion

The root causes and help-seeking need of those across LMICs require attention; the sociocultural experience is nuanced within and between these places. Responsibility for suicidal behaviour needs to be led by LMIC for LMIC to allow for the needs of a particular country, region, and even individual contexts to be understood. To do so efforts to understand the lived experience of those within the country, and regions within is paramount to understanding the barriers to preventing distress leading to suicide.

This systematic review provides insight into the qualitative research published in English exploring the potential causes, consequences, and prevention strategies of suicidal behaviour in LMICs over the past decade. Our study suggests that suicidology needed to be understood culturally, and suicidal behaviour may differ from country to country and even region to region. Additionally, this systematic review showed that suicidal behaviour should be understood and analysed in gendered ways. More research on LMICs is required to come up with versatile solutions to suicidal behaviour.

## Electronic supplementary material

Below is the link to the electronic supplementary material.

Supplementary Material 1

Supplementary Material 2

## Data Availability

Further details of the study and the findings can be provided on request to the corresponding author (Email: hkabir2@une.edu.au).

## Abbreviations

CASP: Critical appraisal skills programme
JBI: Joanna Briggs Institute
LMICs: Lower- and middle-income countries
PRISMA: Preferred reporting items for systematic reviews and meta-analyses
WHO: World health organization
