# Supplementary material for: Qualitative research in suicidology: a systematic review of the literature of low-and middle-income countries
Source: BMC Public Health. 2023 May 19;23:918. doi: 10.1186/s12889-023-15767-9 (PMC10199541; doi:10.1186/s12889-023-15767-9)
Supplement: Supplementary file 1 — Supplementary Material 1 [file 12889_2023_15767_MOESM1_ESM.docx]

**Supplementary File 1. Data base searches, with specific keywords and limiters used for each database.**

| **Database and Search** | **Search Strings and Limiters** | **Results** |
| --- | --- | --- |
| EBSCO Host  ‘All Databases selected’ | AB (suicide OR suicidology OR suiciding OR suicidal)  su(qualitative research OR qual* OR qualitative research design OR narrative OR phenomenology OR interviews OR mixed method)  su(“lived experience” OR prevention OR intervention OR postvention)  Limits applied:  Peer Reviewed  SU and Abstract AB  2011-2021  *All Databases* | **n=964** |
| ProQuest | mainsubject((suicide OR suicidology OR suiciding OR suicidal)) AND su((qualitat* OR "qualitative research design" OR narrative OR phenomenology OR interviews OR "mixed method" OR "mixed methods")) AND mainsubject(("lived experience" OR prevention OR intervention OR postvention))  Limits applied  Peer Reviewed  English  2011-2021 | **n=720** |
| PubMed | Su ((suicide OR suicidology OR suicides OR suicidal OR suiciding) AND  (qualitat* OR "qualitative research design" OR narrative OR phenomenology OR interviews OR "mixed method" OR "mixed methods") AND ("lived experience" OR prevention OR intervention OR postvention))  Filters/Limiters Applied  2010 to 2021 | **n=60** |
| Sage Journals | KEYWORD AND ABSTRACT ((suicide OR suicidology OR suicides OR suicidal OR suiciding) AND (qualitat* OR "qualitative research design" OR narrative OR phenomenology OR interviews OR "mixed method" OR "mixed methods") AND ("lived experience" OR prevention OR intervention OR postvention))  Applied Filters  2010-2021 | **n=564** |
| Web of Science | **suicide OR suicidology OR suicides OR suicidal OR subsiding** (Title) and **qualitat* OR "qualitative research design" OR narrative OR phenomenology OR interviews OR "mixed method" OR "mixed methods"** (Abstract) and **"lived experience" OR prevention OR intervention OR potention** (Title)  Applied Filters  Publication Date: 2010- 2021 | **n=258** |
| Google Scholar | ((suicide OR suicidology OR suicides OR suicidal OR suiciding) AND (qualitat* OR "qualitative research design" OR narrative OR phenomenology OR interviews OR "mixed method" OR "mixed methods") AND ("lived experience" OR prevention OR intervention OR postvention))  Date set to custom range: 2010-2021 | Total Results from Scholar *(3,110)*  Hand selected from first 10 pages of Google Scholar **n=3** |
